# Comparative Analysis of Antioxidant and Anti-Amyloidogenic Properties of Various Polyphenol Rich Phytoceutical Extracts

**DOI:** 10.3390/antiox8010013

**Published:** 2019-01-01

**Authors:** Kody Kleinrichert, Bindhu Alappat

**Affiliations:** Department of Chemistry, Saint Xavier University, Chicago, IL 60655, USA; kleinrichert.k01@mymail.sxu.edu

**Keywords:** Alzheimer’s Disease, amyloid protein, antioxidant, DPPH, *Camellia sinensis*, *Curcuma longa*, *Scoparia dulcis*, catechins, curcuminoids, scoparic acid

## Abstract

Though the pathogenesis of Alzheimer’s Disease (AD) is not completely elucidated, it is generally accepted that the aggregation of toxic amyloid-β (Aβ) protein fibrils plays a major role in the disease’s onset and progression. Various phytoceutical compounds have been shown to attenuate Aβ toxicity and disrupt its aggregation, including various types of polyphenolic compounds. These polyphenolic compounds have also been found to demonstrate potent antioxidant activity, which may contribute to their anti-amyloidogenic properties. This study compares three plants, traditionally used for numerous medicinal purposes in Asian countries, including: *Curcuma longa* (Turmeric), *Camellia sinensis* (Green Tea), and *Scoparia dulcis* (Sweet Broomweed). Antioxidant effects of the crude, polyphenol rich phytoceutical extracts from these plants were analyzed using a 2,2-diphenyl-1-picrylhydrazyl (DPPH) assay. The ability of these extracts to prevent Aβ fibril formation was then carried out in order to establish a relationship between antioxidant activity and Aβ aggregation. A positive correlation between antioxidant efficacy and prevention of Aβ aggregation was demonstrated, indicating that antioxidant activity may play some role in preventing Aβ aggregation.

## 1. Introduction

Alzheimer’s disease (AD) is a non-reversible neurodegenerative disease that slowly impairs brain function, especially in the elderly population. AD is estimated to affect 2.4 million to 4.5 million Americans age 65 and older [1]. It is projected that by 2050 up to 16 million Americans will have the disease [2]. Common symptoms of this disease include: impaired cognitive function, disorientation and confusion, abstract thinking, poor or decreased judgment, loss of normal sleep patterns, learning difficulties, delusions, paranoia, impulsive behavior, and memory loss. The hallmark symptom of AD is the presence of dementia, with the severity varying based on the progression of the disease. Death is a frequent outcome of AD. A recent study that called upon data from the Rush Memory and Aging project found that an estimated 500,000 deaths occurred in 2010 as a result of AD [2]. There is currently no cure for AD and treatments are limited to combating the symptoms. They do nothing in regards to the underlying causes of the disease. 

There are two main neurobiological features of AD present in the brain: neurofibrillary tangles and amyloid plaques. Neurofibrillary tangles are abnormal aggregates of twisted protein fibrils within the neurons. These are caused by the hyperphosphorylation of a protein tasked with the stabilization of neuronal microtubules known as tau. Degradation of tau leads to both the unraveling of neuronal microtubules and the aggregation of the tau protein into “tangles”. These tangles, coupled with the degraded neuronal microtubules, impair the ability of the neurons to communicate with each other [1]. Amyloid plaques, on the other hand, are aggregates of the toxic, insoluble protein Amyloid-β. These plaques form outside neurons and are characteristic of Alzheimer’s Disease. Amyloid-β (Aβ) begins as a membrane protein called amyloid precursor protein (APP). In its normal, non-damaging pathway, APP is first cleaved by alpha secratase then by gamma secratase, forming beneficial proteins to the neurons. However, when beta secratase is the first to cleave the APP, followed by gamma secratase, an Aβ peptide is formed. These proteins aggregate into groups known as Oligomers, which form the amyloid plaques that are characteristic of AD [1]. When aggregated, these amyloid oligomers are largely responsible for the oxidative stress found in the pathogenesis of Alzheimer’s Disease. For this reason, there have been promising results pertaining to the use of antioxidants in the treatment and prevention of Alzheimer’s [3]. Furthermore, many antioxidant compounds have been shown to prevent the formation, extension, and stabilization of Aβ fibrils. It was demonstrated in cell culture experiments that destabilized/degraded Aβ fibrils were less toxic than fully formed fibrils [4]. Antioxidant phytoceuticals are, therefore, a promising avenue of research into the treatment and prevention of Alzheimer’s Disease.

**Figure 1:**
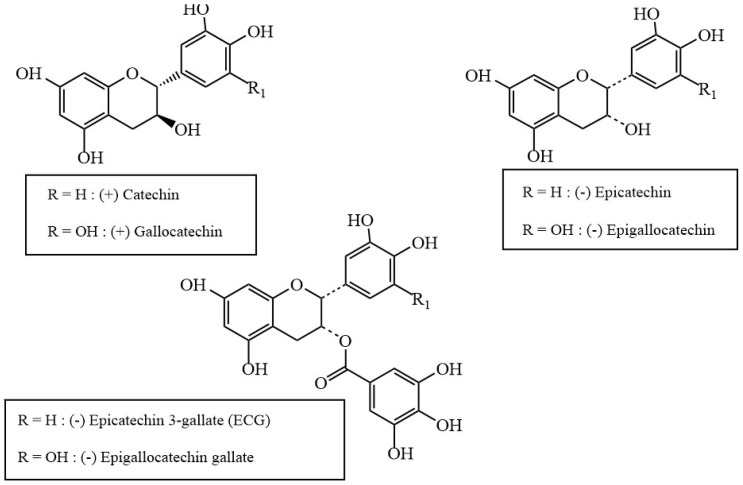
Chemical structures of various catechins found in green tea.

*Camelia sinensis* (*C. sinensis*) is a species of evergreen shrub whose leaves are commonly used to produce different varieties of tea, including green tea. The use of this plant originated in China, several thousand years ago. *C. sinensis* has many compounds that exhibit pharmacological properties; it is therefore exploited for its various health benefits. *C. sinensis* is rich in a variety of polyphenolic compounds collectively known as catechins (Figure 1). According to a study investigating the most efficient method of extraction of the active ingredients of *C. sinensis*, the bioactive compounds in fresh tea leaves consist of around 36% polyphenolic compounds, most of which are catechins [5]. Many studies have been conducted to explore the antioxidant effects of these compounds, including their ability to disrupt amyloid aggregation. One study in particular found that the introduction of (–)-epigallocatechin-3-gallate, the most bioactive catechin found in *C. sinensis*, to Aβ species resulted in the formation of small, unstructured aggregates and mitigated the toxicity of the Aβ species [6]. These catechin compounds are therefore prime candidates for further study regarding their anti-amyloidogenic properties.

**Figure 2:**
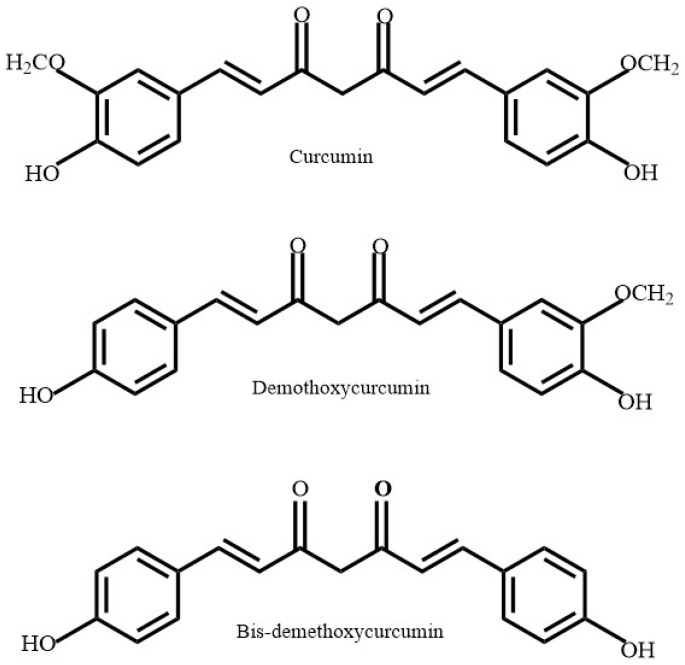
Chemical structure of polyphenols derived from *Curcuma longa*.

Curcuminoids (Figure 2) are another variety of polyphenolic compounds derived from the roots of *Curcuma longa* (*C. longa*), also known as turmeric. *C. longa* has traditionally been used as a spice in many Asian cultures and displays many medicinal properties. The polyphenol rich turmeric spice itself is derived from the dried rhizome of *C. longa*. A study conducted on the neuroprotective effects of curcumin found that it reduced both the amount of oxidative damage and the prevalence of amyloid plaques in a transgenic plaque-forming animal model [7]. The combined documentation of its antioxidant and anti-amyloidogenic properties make it a prime candidate for further comparative analysis to extracts with similar properties.

**Figure 3:**
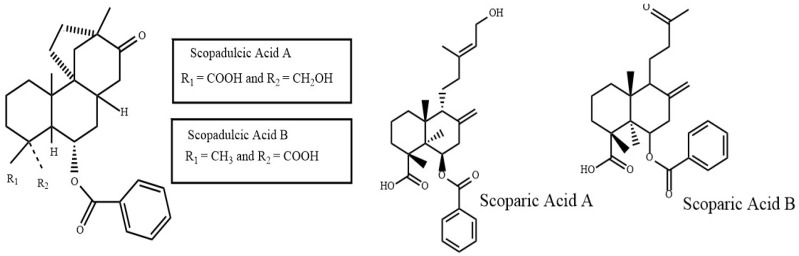
Polyphenols found in *Scoparia dulcis.*

*Scoparia dulcis* (*S. dulcis*), also known as Sweet Broomweed, is an herb frequently found in the tropical and subtropical regions of Southern India. The medicinal effects of *S. dulcis* are well documented amongst various indigenous Indian tribes. It has been used to treat various ailments, including: diabetes mellitus, hypertension, fever, cancer, ulcers, skin lesions and rashes, tuberculosis, and inflammatory issues. This ability is due to its various medicinal properties deriving from its constituent diterpenoid compounds. The compounds within *S. dulcis* that show antioxidant properties are a class of polyphenolic compound known as flavones (Figure 3) [8]. While some research has been conducted on the phytoconstituents of *S. dulcis*, not much has been documented regarding its neuroprotective features, particularly its anti-amyloidogenic properties. 

The goal of this study, therefore, is to establish extraction schemes for the three aforementioned plants with maximal yields of antioxidant polyphenolic compounds. Different extraction schemes were designed, based on a literature review exploring the phytoconstituents and extraction techniques, for each respective plant. The antioxidant activity of these extracts was then analyzed using a 2,2-diphenyl-1-picrylhydrazyl (DPPH) assay. Antioxidant efficacy was then compared to the anti-amyloidogenic potency of the extract using a thioflavin T (ThT) Amyloid-β aggregation assay.

## 2. Materials and Methods

### 2.1. Materials Section

Green tea samples were purchased from Bigelow Tea Company and were used without further processing. Powdered curcumin samples were obtained from House of Spices, Inc. and used, as received, for extraction. Fresh leaves of *Scoparia dulcis* were collected from the neighborhood of Kottayam, Kerala, India and were air dried before being powdered. The powdered samples were kept in air-tight polyurethane bags at room temperature. All solvents used in the experiment—acetone, methanol, hexane, and chloroform—were purchased from Sigma Aldrich Chemicals. For the antioxidant analysis, DPPH was obtained from Alfa-Aesar Co., Inc. and the microplates were from Fisher Scientific. A SensoLyte thioflavin T (ThT) Amyloid-β aggregation assay kit was purchased from Anaspec and used to study the Amyloid-β (Aβ) aggregation.

### 2.2. Phytoceutical Extraction

Different extraction schemes were designed according to the literature review. The goal of each respective extraction was to maximize the amount of antioxidant polyphenols extracted.

The extraction of catechins from *C. sinensis* was carried out using 50% aqueous acetone and a boiling time of 2 h. This solvent and its respective conditions were found to yield maximum catechins with minimum degradation [5]. Next, 80 mL of 50% aqueous acetone was combined with approximately 4.000 g of commercially purchased, ground *C. sinensis*. The solution was heated with a ceramic hot plate in a 125 mL Erlenmeyer flask with a magnetic stir bar for two hours. The flask was covered with a watch glass to minimize solvent loss. Small aliquots of 50% aqueous acetone were used to rinse down tea leaves that clung to the sides of the flask due to boiling. After two hours, the watch glass was removed and the excess acetone boiled off. The remaining aqueous solution was then separated from the solid plant material by vacuum filtration. The resulting solution was then partitioned with two 40 mL aliquots of hexanes, to remove lipid components, and two 40 mL aliquots of chloroform, to remove caffeine [9]. The partitioned solvent fractions were discarded and the aqueous phase was transferred to a previously weighed 50 mL beaker. The excess solvent was boiled off using a hot plate under a steady stream of nitrogen. The beaker was then weighed again. The mass of the original beaker was subtracted from this value to give the mass of phytoceutical compounds extracted. These compounds were re-suspended in methanol at a volume of 1 mg/mL.

The extraction of curcuminoids was carried out, using pure acetone as the solvent, at room temperature for three hours [10]. Then, 4.000 g of commercially purchased, powdered *C. longa* was combined with 80 mL of 99.5% reagent grade acetone and allowed to sit while being stirred with a magnetic stir bar for 3 h. The solution was then vacuum filtered, to remove the solid plant material, and transferred to a previously weighed 100 mL beaker. The solvent was evaporated using a hot plate under a steady stream of nitrogen and the beaker was reweighed. The mass of the original beaker was subtracted from this value to give the mass of phytoceutical compounds extracted. These compounds were re-suspended in methanol at a volume of 1 mg/mL.

The extraction of flavones from *S. dulcis* was carried out in a manner identical to the catechin extraction from *C. sinensis*. This was so, because catechins and flavones belong to a family of polyphenolic compounds known as flavonoids. Because of the structural retention between the two compounds, the same extraction technique could be utilized for *S. dulcis.* For this, 80 mL of 50% aqueous acetone was combined with approximately 4.000 g of dried, ground *S. dulcis* personally attained in India. The solution was heated with a ceramic hot plate in a 125 mL Erlenmeyer flask with a magnetic stir bar for two hours. The flask was covered with a watch glass to minimize solvent loss. Small aliquots of 50% aqueous acetone were used to rinse away plant matter that had clung to the sides of the flask during boiling. After two hours, the watch glass was removed and the excess acetone boiled off. The remaining aqueous solution was then separated from the solid plant material by vacuum filtration. The remaining solution was then partitioned with two 40 mL aliquots of hexane, to remove lipid components, and two 40 mL aliquots of chloroform, to remove caffeine [9]. These organic fractions were discarded and the aqueous phase was transferred to a previously weighed 50 mL beaker. The remaining solvent was boiled off using a sand bath under a steady stream of nitrogen and the beaker was weighed again. The mass of the original beaker was subtracted from this value to give the mass of phytoceutical compounds extracted. These compounds were re-suspended in methanol at a volume of 1mg/mL.

### 2.3. DPPH Analysis

**Figure 4:**
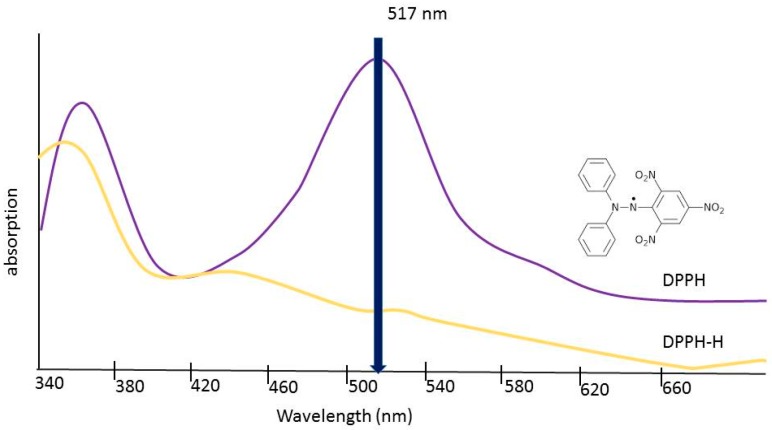
DPPH absorption spectra.

Eight dilutions, each of 1 mg/mL stock solutions, were prepared with methanol at concentrations of 100 μg/mL, 200 μg/mL, 300 μg/mL, 400 μg/mL, 500 μg/mL, 600 μg/mL, 800 μg/mL, and 1000 μg/mL. Stock 2,2-diphenyl-1-picrylhydrazyl (DPPH) was prepared by combining 0.0025 g of powdered DPPH with 100 mL of methanol in an Erlenmeyer flask. The methanol was measured using a graduated cylinder. The flask was covered with aluminum foil to prevent photobleaching of the compound and the solution was stirred with a magnetic stir bar for 10 min. Next, 5 μL of each dilution of the three extracts were combined with 195 μL of DPPH solution in a 96-well microplate. After, 5 μL of methanol was also combined with the DPPH solution to act as a negative control. To normalize the data, by factoring out the absorbance value caused by the extract itself, 5 μL of the same dilutions were combined with methanol in separate wells. The 5 μL of the test sample were first added to the microplate with a micropipette. Then, 195 μL of DPPH solution or pure methanol was then added to the appropriate wells using a multichannel pipette. Samples were covered to prevent exposure to light and then allowed to incubate at room temperature for 15 min. As seen in Figure 4, the radical form of DPPH absorbs light at 515 nm but the reduced form does not. The absorbance of each well was read using an absorbance microplate reader (Biotek) at 515 nm to detect any reduction of the DPPH free radical [11]. 

Statistical analysis was carried out using Microsoft Excel 2011. Four replicates of each test were run, in which the compound and dilution were combined with DPPH. The absorbance of the dilution of each test compound, combined with pure methanol, was subtracted from the absorbance of each corresponding well with DPPH and the respective test compound dilution. The corrected absorbance values were then subjected to the Grubbs Test for outliers according to the following formula:

(1) $$G_{calc}=\frac{\left|PreliminaryMean-QuestionableValue\right|}{PreliminaryStandardDeviation}$$

If the calculated value of G ($G_{calc}$) was greater than the standard value of G for four replicates ($G_{Table}$) of 1.46 then the value was rejected as an outlier at 95 percent confidence. The nonrejected data was used to calculate the mean absorbance for each test compound dilution and the respective negative control. Percent inhibition of DPPH was then calculated according to the following formula:

(2) $$\%Inhibition=\left(\frac{AB_{i}-AB_{f}}{AB_{i}}\right)\times 100$$

where ($AB_{i}$) is the initial absorbance of DPPH, attained using the measured absorbance value of DPPH and pure methanol, and ($AB_{f}$) is the final absorbance of DPPH, attained using the measured absorbance value of DPPH and the respective test compound dilution. A graph of the extract concentration vs. the percent inhibition was then constructed.

### 2.4. Amyloid Aggregation Analysis

Amyloid aggregation analysis was carried out using a SensoLyte thioflavin T Amyloid-β (1–42) aggregation kit (Anaspec). First, 10 μL of 20 mM thioflavin T (ThT) stock solution was combined with 90 μL of assay buffer in order to dilute it to a 2 mM working solution. Then, 1000 μL of assay buffer were added to the dehydrated Amyloid-β (Aβ) peptide. This solution was mixed by inversion until all of the peptide had dissolved. Next, 85 μL of Aβ and 10 μL of 2 mM ThT were added to each well of interest in a black fluorescence 96-well microplate (Fisher Scientific). After, 5 μL of methanol were added to the first well. This served as a positive control as nothing was added to inhibit Aβ aggregation. Subsequently, 5 μL of each extract, at a concentration of 500 μg/mL, was added to the wells. Finally, 5 μL of 2 mM Morin, a known inhibitor of Aβ aggregation, was added to the last well. This served as a negative control for the assay, signifying high disruption of Aβ aggregation. The plate was incubated at 37 °C for two hours and shaken intermittently in order to facilitate aggregation. Well fluorescence was then read using a Synergy 2 multi-mode reader (Biotek) with excitation at 440 nm and emission at 485 nm. The sensitivity was automatically scaled to 216 for the first trial and manually set at 216 for all of the subsequent trials. Four replicates were performed for each extract. Due to a lack of resources, only 3 replicates were performed for the positive and negative controls.

Statistical analysis was carried out in Microsoft Office Excel 2011. No values were rejected as outliers according to the Grubbs Test. The mean and standard deviation were calculated for each test compound or extract. The results were then graphically represented with a bar graph. Error bars were added, showing uncertainty of plus or minus one standard deviation.

## 3. Results

**Figure 5:**
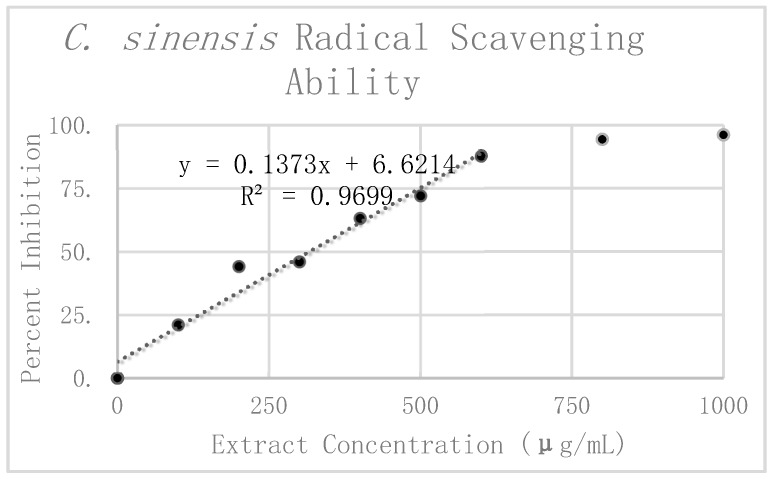
DPPH assay results and radical scavenging effects of *C. sinensis*.

Results of DPPH analysis for *C. sinensis* can be seen in Table 1. The preliminary mean and standard deviation were calculated using the raw absorbance values from the four *C. sinensis* replicates. The Grubbs test for outliers was then performed on the value furthest from the preliminary mean for each concentration. One value was rejected at 300 μg/mL. The corrected mean and standard deviation were then calculated for this concentration. Percent inhibition was calculated according to the corrected mean absorbance. These values are graphically displayed in Figure 5. Linear regression analysis was used to find the slope of the best fit line as a relative way to compare the antioxidant potency of the extracts. This linear regression analysis was performed on the first six dilutions up to 600 μg/mL. Beyond 600 μg/mL the extract reached nearly complete inhibition, causing it to break from the expected linear trend. For this reason, extracts at concentrations of 800 μg/mL and 1000 μg/mL were excluded from the line of best fit. All in all, the linear regression analysis resulted in a line of best fit with a slope of 0.1526 and an R^2^ value of 0.95254.

**Figure 6:**
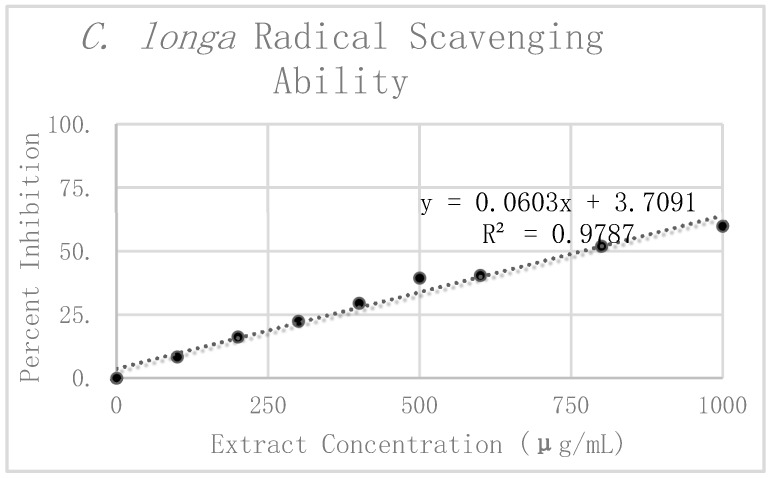
DPPH analysis and radical scavenging ability of *C. longa*.

The results of the DPPH analysis for *C. longa* are summarized in Table 2. The preliminary mean and standard deviation were calculated using the raw absorbance values of the four *C. longa* replicates. The Grubbs test for outliers was then performed on the value furthest from the preliminary mean for each concentration. Two values were rejected, one at 300 μg/mL and the other at 1000 μg/mL. The corrected means and standard deviations were then calculated for these concentrations. Percent inhibition was calculated according to the corrected mean absorbance and are also presented graphically in Figure 6. Linear regression analysis was again used to find the slope of the best fit line as a relative way to compare the antioxidant potency of the extracts. The linear regression analysis resulted in a line of best fit with a slope of 0.066 and an R^2^ value of 0.96562.

**Figure 7:**
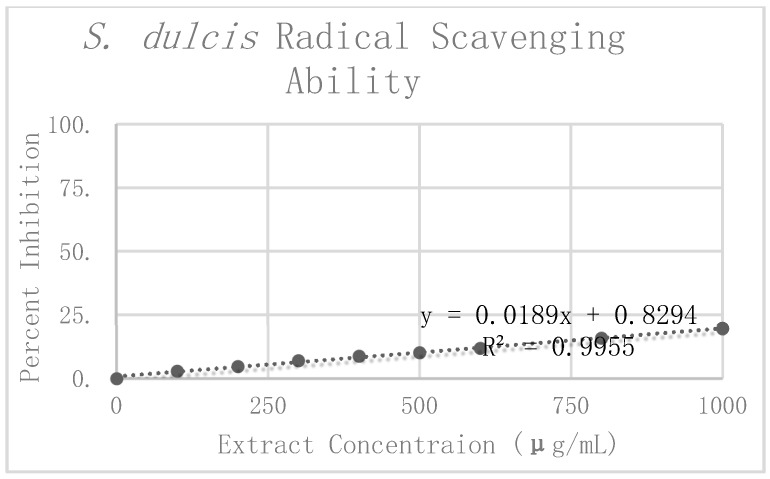
DPPH assay results and radical scavenging effects of *S. dulcis*.

In Table 3, the preliminary mean and standard deviation were calculated using the raw absorbance values from the four *S. dulcis* replicates. The Grubbs test for outliers was then performed on the value furthest from the preliminary mean for each concentration. One value was rejected at 100 μg/mL. The corrected means and standard deviations were then calculated for this concentration. Percent inhibition was calculated according to the corrected mean absorbance. These values can be seen graphically in Figure 7. Linear regression analysis was again used to find the slope of the best fit line as a relative way to compare the antioxidant potency of the extracts. The linear regression analysis resulted in a line of best fit with a slope of 0.0202 and an R^2^ value of 0.9888.

When comparing the antioxidant potency of the respective extracts, we found that the higher the value of the slope, the less concentrated the extract needed to be to exhibit the same radical scavenging ability. In using the slope of the linear trend lines in Figure 4, Figure 5, and Figure 6 as a means to compare antioxidant potency, we saw that *C. sinensis* displayed the most antioxidant activity, with a slope of 0.1526. This was followed by *C. longa* and *S. dulcis*, with respective slopes of 0.066 and 0.0202. From these results, the concentration of 500 μg/mL was selected for each of the extracts for use in amyloid aggregation analysis. This concentration adhered closely to the line of best fit for all three extracts and did not reach complete inhibition in *C. sinensis*.

**Figure 8:**
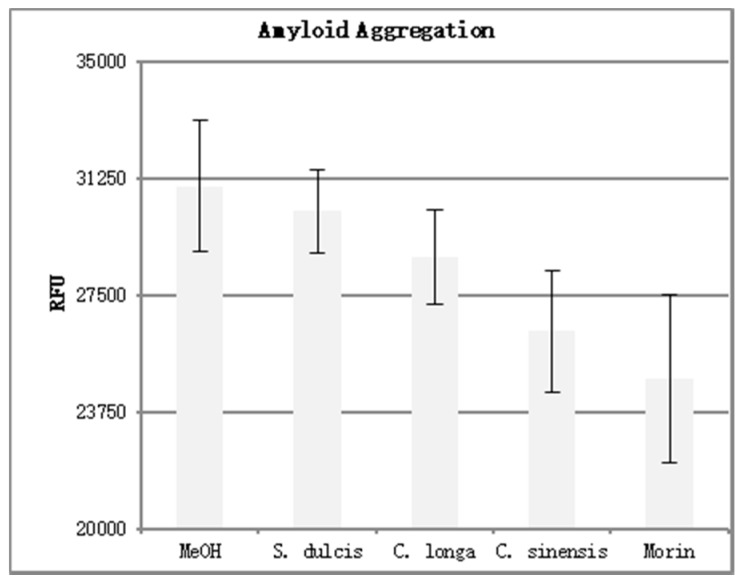
Amyloid aggregation assay results.

The results of the amyloid aggregation assay can be seen in Table 4. Four replicates were performed for each phytoceutical extract at a concentration of 500 μg/mL. Three replicates were performed for the positive and negative controls. The mean fluorescence was calculated and is shown in graph form in Figure 8. Error bars were added at one standard deviation above and below the mean.

Higher fluorescence values (RFU) indicate an increase in Aβ aggregation. Morin, a known inhibitor of Aβ aggregation, displayed the lowest fluorescence of the compounds tested. This indicates the most disruption of Aβ aggregation. When analyzing the three extracts for their ability to inhibit aggregation, we see that *C. sinensis* proved to be the most effective, followed by *C. longa* and *S. dulcis* respectively. This mirrored the results seen in the DPPH analysis, with the most potent antioxidant extract (*C. sinensis*) causing the most Aβ aggregation inhibition, and the least potent antioxidant extract (*S. dulcis*) causing the least Aβ aggregation inhibition.

## 4. Discussion

The extraction techniques were carried out based on previously established techniques found in the literature. In a study comparing various solvents in the extraction of catechins from *C. sinensis,* 50% aqueous acetone boiled for 2 h yielded maximum extraction with minimal degradation [5]. Another study found that the most efficient way to remove lipid compounds and caffeine from *C. sinensis* extracts was via partitioning with hexanes and chloroform [9]. Combining these general principles allowed for the efficient extraction of catechins from *C. sinensis.* Extracts from *S. dulcis* were prepared using the same extraction scheme as the chemical properties of the flavone compounds isolated in *S. dulcis* are similar to the catechin compounds present in *C. sinensis.* The *C. longa* extraction was carried out according to a study that examined the most efficacious solvents for extracting curcuminoids from the raw plant material. It identified pure acetone at room temperature for 3 h to be the best solvent [10]. The solvent used to prepare these extracts was subsequently removed and the resultant phytoceutical compounds were re-suspended at a standard concentration, for comparative purposes. While qualitative analysis was not carried out on the final solutions, based upon our literature review, we feel it is within reason to assume they contain the compounds of interest.

The positive correlation between antioxidant efficacy and disruption of Aβ aggregation leads us to believe that the antioxidant activity of these polyphenols may be responsible for disrupting Aβ aggregation. While it has been widely hypothesized that antioxidant species could exhibit neuroprotective effects in the prevention of oxidative neuronal damage in AD, these results suggest a method by which they may carry out this protective role.

Metallic cationic species, specifically Cu, Fe, and Zn, have been proven to play a role in the formation of Aβ plaques in AD. While their exact role in the formation of fibrils and plaques in vivo is unclear, it is clear that they induce aggregation due to their concentrated presence in and around Aβ plaques. Because of their ionic nature, these species can participate freely in redox reactions. Interestingly enough, Aβ aggregation actually catalyzes the reduction of Cu^2+^ to Cu^+^ and Fe^3+^ to Fe^2+^ [12]. It is our hypothesis that the premature reduction of these cations by the antioxidant polyphenolic compounds present in the extracts causes a disruption in the mechanism of Aβ aggregation. This would, in turn, cause a marked decrease in Aβ aggregation, preventing senile plaque formation in AD patients. Furthermore, it was found that Aβ needs to be aggregated to exhibit neurotoxic effects [4]. Therefore, the disruption of Aβ aggregation also plays a role in the prevention of the oxidative stress inherent to the pathogenesis of AD.

The oxidative stress present in AD patients has various causes; however, it has been shown that this oxidative damage is highly concentrated around Aβ plaques. The reduction of Cu and Fe by Aβ results in the generation of hydrogen peroxide. These H_2_O_2_ molecules, if not properly cleared from the body, can further react to produce reactive oxygen species (ROS) [12]. Antioxidant compounds, such as the polyphenols present in the aforementioned phytoceutical extracts, can also function to reduce these free radicals, preventing oxidative neuronal stress.

Given that antioxidant compounds can function to not only remedy some of the oxidative damage present in AD but may also prevent Aβ aggregation, it can be suggested that they may provide a novel method for both prevention and treatment of AD.

## 5. Conclusions

The results of this study indicate a positive correlation between antioxidant efficacy and the disruption of Aβ aggregation. This means, that as antioxidant activity increased, Aβ aggregation decreased. The most potent antioxidant extract (*C. sinensis*) caused the most Aβ aggregation inhibition. The second most potent antioxidant extract (*C. longa*) caused second most Aβ aggregation inhibition. Finally, the least potent antioxidant extract (*S. dulcis*) caused the least Aβ aggregation inhibition. While it is evident that there was a positive correlation between antioxidant potency and the extent of Aβ aggregation inhibition, further study with more antioxidant compounds is needed to further elucidate if this is a true cause and effect relationship.

## Figures and Tables

**Table 1:** DPPH assay raw absorbance data and calculated values for *C. sinensis*.

Concentration	0 μg/mL	100 μg/mL	200 μg/mL	300 μg/mL	400 μg/mL	500 μg/mL	600 μg/mL	800 μg/mL	1000 μg/mL
Preliminary Mean	0.379	0.299	0.212	0.181	0.139	0.106	0.046	0.022	0.015
SD	0.0050	0.0070	0.0367	0.0479	0.0161	0.0369	0.0038	0.0030	0.0010
Grubbs Value	1.40	1.29	1.36	1.46	1.24	1.19	1.32	1.33	1.00
Corrected Mean	0.379	0.299	0.212	0.204	0.139	0.106	0.046	0.022	0.015
Corrected SD	0.0050	0.0070	0.0367	0.0132	0.0161	0.0369	0.0038	0.0030	0.0010
Percent Inhibition	0.00	21.17	44.20	46.09	63.26	72.16	87.80	94.33	96.11

**Table 2:** DPPH assay raw absorbance data and calculated values for *C. longa*.

Concentration	0 μg/mL	100 μg/mL	200 μg/mL	300 μg/mL	400 μg/mL	500 μg/mL	600 μg/mL	800 μg/mL	1000 μg/mL
Preliminary Mean	0.409	0.375	0.343	0.316	0.288	0.248	0.243	0.196	0.159
SD	0.0037	0.0043	0.0089	0.0039	0.0061	0.0156	0.0262	0.0152	0.0105
Grubbs Value	1.35	1.40	1.24	1.53	0.98	1.22	1.18	1.45	1.52
Corrected Mean	0.409	0.375	0.343	0.317	0.288	0.248	0.243	0.196	0.164
Corrected SD	0.0037	0.0043	0.0089	0.0015	0.0061	0.0156	0.0262	0.0152	0.0010
Percent Inhibition	0.00	8.37	16.26	22.41	29.52	39.49	40.53	52.02	59.90

**Table 3:** DPPH assay raw absorbance data and calculated values for *S. dulcis*.

Concentration	0 μg/mL	100 μg/mL	200 μg/mL	300 μg/mL	400 μg/mL	500 μg/mL	600 μg/mL	800 μg/mL	1000 μg/mL
Preliminary Mean	0.386	0.376	0.368	0.359	0.352	0.347	0.340	0.325	0.310
SD	0.0022	0.0027	0.0006	0.0050	0.0050	0.0026	0.0090	0.0044	0.0049
Grubbs Value	1.36	1.48	0.83	1.40	1.00	1.34	1.11	1.36	1.22
Corrected Mean	0.386	0.375	0.368	0.359	0.352	0.347	0.340	0.325	0.310
Corrected SD	0.0022	0.0006	0.0006	0.0050	0.0050	0.0026	0.0090	0.0044	0.0049
Percent Inhibition	0.00	2.94	4.79	7.06	8.87	10.23	11.92	15.93	19.62

**Table 4:** Amyloid aggregation assay raw fluorescence data and calculated values.

Sample	MeOH	*S. dulcis*	*C. longa*	*C. sinensis*	Morin
Trial 1	29531	30691	28, 682	24,267	22,805
Trial 2	30,037	31,488	30,818	27, 628	27,869
Trial 3 (Replicate 1)	33,404	30,233	28,066	28, 297	23,777
Trial 3 (Replicate 2)		28,313	27,252	25,096	
Mean	30,990	30,181	28,705	26,322	24,817
Standard Deviation	2105	1349	1526	1944	2687
